# The impact of elective physical education on high school students' core PE competencies: a quasi-experimental study

**DOI:** 10.3389/fpsyg.2026.1726417

**Published:** 2026-03-26

**Authors:** Xiaotian Wang, Binping Gong, Linqin Song, Mingming Guo

**Affiliations:** 1 Department of Physical Education, College of Education for the Future, Beijing Normal University, Zhu Hai, China; 2 College of Physical Education and Sport, Beijing Normal University, Beijing, China

**Keywords:** China, core competencies in physical education, elective physical education, high school students, quasi-experimental

## Abstract

**Background:**

Elective physical education (EPE) is a key curricular reform in China, yet evidence on its influence on high school students' core competencies in physical education (CCPE) remains limited. This study examined whether EPE was associated with greater pre–post improvements in CCPE among Chinese high school students.

**Methods:**

A quasi-experimental, school-level allocated, parallel-group pretest–posttest design was conducted with 196 Grade 11 students in Chongqing, China. The intervention group received EPE, and the control group continued conventional PE. CCPE was assessed using the Core Competencies in Physical Education Evaluation Scale for High School Students. Intervention effects were tested using a 2 (time) × 2 (group) mixed-design repeated-measures ANOVA.

**Results:**

Significant time × group interactions were observed for overall CCPE and all three domains (athletic ability, health behaviors, and sport morals), indicating small but consistent differential pre–post gains favoring EPE relative to conventional PE in students' self-reported (perceived) competencies.

**Conclusion:**

Under routine school conditions, EPE was associated with small improvements in students' CCPE as measured by self-report. Given the quasi-experimental design, findings should be interpreted as associations, and residual confounding cannot be ruled out even with baseline equivalence. Future studies should incorporate longer follow-up and objective or observational outcomes.

## Introduction

Core competencies in physical education (CCPE) are recognized as culturally contingent. In recent years, this competency-based orientation has been incorporated into national PE standards in multiple countries. For example, the U.S. National Standards for Physical Education define CCPE as “the ability and confidence to engage in a variety of physical activities across multiple environments that promote the healthy development of the whole person” (SHAPE America - Society of Health Physical Educators, 2024). Similarly, the Australian Physical Education Curriculum defines CCPE as “the capacity of individuals to access, comprehend, and apply information to maintain and enhance health and wellbeing” (Australian Curriculum Assessment Reporting Authority [ACARA], 2012). Although these frameworks share broad emphases (e.g., participation, health, and socio-moral development), definitions and assessment practices remain context dependent, underscoring the need for evidence generated within specific curriculum systems to inform implementation and scale-up.

In China, the General Senior High School Physical Education Curriculum Standards (2017) and the Physical Education Curriculum Standards for Compulsory Education (2022) position disciplinary core competencies as central to the educational value of PE. The standards emphasize that students develop these competencies progressively through PE learning, encompassing values, dispositions, and key capabilities. Within this policy framework, CCPE comprises three interrelated domains: athletic ability (AA), health behaviors (HB), and sport morals (SM). AA refers to the integrated expression of physical fitness, technical-tactical proficiency, and psychological readiness in physical activity and is regarded as foundational to movement performance. HB reflects the capacity to maintain and enhance physical and mental wellbeing while adapting to environmental demands. SM concerns the behavioral norms and ethical principles guiding sport participation, together with the value orientations and dispositional qualities cultivated through sporting practice (Ministry of Education of the People's Republic of China., 2018, 2022).

CCPE are widely viewed as a key indicator of achieving PE objectives and an important lever for curriculum reform and quality improvement (Hogan et al., 2023; Carl et al., 2022). Empirical studies suggest that stronger CCPE is associated with more positive attitudes toward PE and sport, greater interest in skill development, and higher participation in physical activity (Mazzoli et al., 2024). CCPE has also been linked to healthier lifestyle patterns and more adaptive self-regulatory processes in PE, such as goal-setting, planning, and self-monitoring (Giblin et al., 2014; Ma et al., 2024; Wang and Chen, 2022). Collectively, CCPE can be conceptualized as both a curricular outcome and a proximal set of capacities shaping students' engagement and learning trajectories in PE.

PE is a central vehicle for developing CCPE, providing curriculum-aligned contexts in which students can build AA, HB, and internalize SM (Wijaya et al., 2025). Consistent participation in PE is also associated with competencies that support lifelong physical activity and both physical and mental health (Mahindru et al., 2023; Piñeiro-Cossio et al., 2021). However, PE is implemented through diverse instructional organizations and pedagogical approaches, and features such as student choice, grouping structures, practice progression, and feedback may shape AA-, HB-, and SM-related outcomes in different ways—highlighting the importance of examining curriculum models that systematically vary these learning opportunities.

Furthermore, elective physical education (EPE) has been proposed as a curriculum-organization approach to support the multidimensional development of CCPE. In contrast to conventional, teacher-directed PE, EPE institutionalizes meaningful choice by allowing students to enroll in an interest-aligned sport module for a sustained period, which has been linked to greater engagement, skill learning, and motivational benefits when implemented with fidelity (Gao, 2025; O'Connor, 2022; Hernando-Garijo et al., 2021). Emerging comparative evidence also suggests potential advantages for health-related outcomes and athletic ability relative to traditional formats (Qianqian et al., 2023). Conceptually, these features align with autonomy-supportive pedagogy and may better support students' autonomy needs, with downstream implications for broader CCPE development (Fernandez-Rio and Iglesias, 2024). In this study, EPE refers to a timetabled, curriculum-aligned elective structure within mandatory PE in Chinese upper-secondary schools, where students choose from parallel sport modules and are taught in cross-class elective groups rather than fixed homeroom classes. This institutionalized arrangement differs from more loosely defined “choice-based” approaches that primarily involve within-class task choice or extracurricular options.

Despite growing interest, the evidence base on EPE remains limited in three key respects. First, most studies are cross-sectional or descriptive, offering limited leverage for judging whether EPE is associated with greater growth in CCPE than conventional PE. Second, prior work has focused mainly on readily quantifiable outcomes (e.g., AA, fitness, or participation), with comparatively less attention to HB and SM—both central to China's CCPE framework and policy priorities (Dai et al., 2022). Third, even when elective or choice-based models are examined, the instructional mechanisms linked to multidimensional CCPE development are often assumed rather than explicitly theorized or tested. Collectively, these gaps leave uncertainty for schools implementing EPE amid ongoing curriculum reform and limit evidence-informed scale-up.

Self-Determination Theory (SDT) provides a useful framework for articulating how EPE may be associated with AA, HB, and SM. SDT posits that autonomy, competence, and relatedness support foster internalized motivation and adaptive learning and behavioral outcomes (Ryan and Deci, 2000). In an EPE context, meaningful sport choice can support autonomy, structured practice and feedback can support competence (most directly relevant to AA), and stable elective groups with cooperative tasks can support relatedness through belonging and mutual accountability (relevant to SM). Together, these need-supportive experiences may also promote the internalization of health-oriented values and self-regulatory skills that underpin HB.

China's recent PE curriculum reform has mandated elective courses at the high school level, yet evidence on the real-world effectiveness of EPE in Chinese high schools remains limited. Using a quasi-experimental, school-level pretest-posttest design with a conventional PE comparison group, we examined whether EPE is associated with greater improvements in (a) overall CCPE and (b) each CCPE domain (AA, HB, and SM). Guided by SDT and prior research on choice-based PE, we hypothesized that students receiving EPE would show larger gains in overall CCPE and in each domain, with potentially varying magnitudes across domains. This study provides policy-relevant evidence to inform the implementation and refinement of EPE within China's ongoing curriculum reform.

## Methods

### Research design

This study employed a quasi-experimental, school-level allocated, parallel-group pretest–posttest design. One public high school implemented an EPE program for 12 weeks, while a comparable public high school continued conventional PE. CCPE (overall and three domains: AA, HB, and SM) was assessed via standardized questionnaires at pretest and posttest. Intervention effects were examined using a 2 (time: pretest vs. posttest) × 2 (group: EPE vs. control) mixed-design repeated-measures ANOVA. Given the non-randomized, school-level allocation, findings are interpreted as associations rather than definitive causal effects.

### Participants

A total of 196 Grade 11 students participated. The intervention group comprised 94 students from Bashu High School in Chongqing (48 male, 46 female), and the control group comprised 102 students from Chengnan High School (51 male, 51 female) (Table 1). Eligibility criteria required that students be able to participate fully in routine PE classes. Both schools are public urban high schools located in Chongqing and follow the national senior high school curriculum standards, including regular PE class arrangements. Both schools are under the same municipal education administration and implemented comparable PE timetables and curriculum standards, providing a pragmatic basis for treating the control school as a reasonable counterfactual under real-world constraints. Nevertheless, unobserved school-level factors (e.g., school culture, teacher characteristics, or extracurricular sport opportunities) may differ between sites and cannot be fully ruled out. The participating students' gender was self-reported (male/female) and was comparable across groups and is consistent with typical Grade 11 cohorts in urban public high schools in Chongqing. Because the sample was drawn from only two schools in one city, the findings should not be interpreted as nationally representative. Guardian permission and written informed consent were obtained from all participants.

**Table 1:** The participants.

**Group**	**Intervention group**	**Control group**
**Male (N)**	48	51
**Female (N)**	46	51

### Procedure

The study received ethical approval from the Ethics Review Committee of Beijing Normal University. Prior to data collection, a member of the research team who also served as a teacher obtained authorization from the principals of Bashu High School and Chengnan High School in Chongqing to conduct the intervention. Following approval by the instructional affairs committees of both schools, a subset of Grade 11 students was recruited to participate in the study. All procedures complied with applicable institutional and national guidelines governing research involving human participants.

A 12-week EPE program was delivered to the intervention group. At baseline, students selected an interest-based elective that determined the sport-specific curriculum they would follow during the intervention. Options included basketball, soccer, pickleball, aerobics, and other sports; selections remained fixed for the 12-week intervention. Because sport selection was interest-based, we acknowledge that this procedure may introduce self-selection (e.g., students with higher initial motivation or prior sport experience may preferentially select certain modules). To mitigate this risk, all elective modules followed a common instructional framework with identical contact time, shared learning objectives, and standardized assessment procedures, and the overall implementation was monitored throughout the intervention.

The control group continued to receive conventional PE classes typically delivered in Chinese K−12 schools. Instruction for the control group followed the standardized content prescribed by the General High School Physical Education Curriculum Standards and was organized according to the schools' existing academic class structure.

CCPE was assessed both as a composite construct and across its subdomains (AA, HB, and SM) at baseline and post-intervention in both intervention and control groups. All measures were collected via self-administered questionnaires that students completed independently. Questionnaires were administered in a supervised classroom setting during regular school hours, following a standardized script delivered by trained evaluators. Students were seated apart and instructed not to discuss their answers. Responses were collected anonymously to reduce evaluation-related pressure, and teachers were not involved in scoring or viewing individual responses. The completion time was approximately 15 min, and questionnaires were checked on-site for missing items before submission. Baseline equivalence between groups was examined on key outcomes prior to the intervention; nevertheless, unmeasured differences in motivation or prior skill cannot be fully ruled out and are addressed as a limitation.

### Intervention and fidelity

#### Intervention design

The study was conducted over a 12week intervention delivered between November 2024 and March 2025 at Bashu and Chengnan High Schools in Chongqing, China. Before the intervention, both schools followed the high school PE timetable and organization in China: three 45 min lessons per week delivered to fixed homeroom classes, with content prescribed by the General High School Physical Education Curriculum Standards (e.g., track and field, ball sports). Consistent with the Standards, this teacher-directed approach emphasized physical conditioning and standardized skill instruction while allowing limited student autonomy in activity selection. To enhance feasibility and implementation fidelity, the research team and PE teachers at both sites co-developed teaching plans and detailed procedures for each study arm. In this study, EPE was implemented as a school-scheduled, timetabled elective module within the regular PE curriculum, featuring cross-class regrouping and fixed module enrollment across the full 12-week period, which distinguishes it from within-class choice or short-term rotation formats commonly described as choice-based PE.

The intervention group received an EPE model consisting of three 45 min sessions per week; students selected a single sport module (e.g., basketball, aerobics, pickleball, or ultimate frisbee) based on personal interest. A defining feature of the EPE model was the removal of fixed-class boundaries: students were regrouped into cross-class elective modules to expand choice. For clarity and replicability, EPE in this study is conceptualized as a choice-based, autonomy-supportive instructional model characterized by sustained module enrollment, progressive skill learning, and competence-oriented feedback aligned with CCPE targets. In addition to cross-class regrouping, EPE emphasizes meaningful choice, stable peer learning communities, and structured practice progression within a selected sport module, which distinguishes it from conventional, rotation-based, teacher-directed PE. To reduce systematic variation across modules that could confound outcomes, each sport module was delivered using a standardized lesson structure (warm-up, skill development, structured practice/game play, cool-down), aligned competency targets, and common assessment criteria. Teachers received unified training and used harmonized teaching plans developed jointly by the research team and school PE staff.

The control group continued conventional PE on the same schedule, with standards-aligned instruction delivered within fixed homeroom classes; content included mandatory physical conditioning (e.g., 1,000-meter runs, push-ups) and prescribed sport-specific drills (e.g., high- and low-dribbling and moving shots in basketball; instep kicking in soccer).

Contact time, facility access, and equipment availability were held constant across groups; instructional organization was the primary planned difference between conditions. To strengthen internal validity, both groups were assessed using the same CCPE instrument and a common scoring rubric. Baseline and post-intervention assessments were administered by an evaluation team that received centralized training and followed standardized protocols. Because students were not randomized at the individual level and sport choice was voluntary within the intervention condition, causal interpretations are made cautiously, and potential self-selection is explicitly considered in the Discussion.

#### Fidelity

To ensure standardized delivery of the intervention, a systematic implementation fidelity framework was established. During the 12 week intervention, the third author provided on-site supervision of daily PE, and lesson delivery and student progress were systematically documented. Weekly implementation meetings with PE teachers at both schools were convened by the first and second authors to provide structured feedback, address emergent issues in real time, and maintain detailed minutes and process records, thereby creating an implementation audit trail.

#### Tools and measurements

Students' CCPE were assessed using the Core Competencies in Physical Education Evaluation Scale for High School Students, an instrument that has demonstrated acceptable reliability and validity in samples of Chinese high school students (Dai, 2023), in which the scale demonstrated excellent overall internal consistency (Cronbach's α = 0.94) and good subscale reliability (AA α = 0.89; HB α = 0.93; SM α = 0.79), with sampling adequacy for factor analysis acceptable to meritorious (Kaiser–Meyer–Olkin overall = 0.72; AA = 0.87; HB = 0.85; SM = 0.76), supporting the instrument's reliability and construct validity. The instrument comprises 45 items organized into three validated subscales (AA, HB, and SM); the full item wording is provided in the Supplementary Questionnaire (see Supplementary material). All items were rated on a five-point Likert scale (1 = strongly disagree to 5 = strongly agree). Subscale scores were computed as the mean of their constituent items; higher values indicate stronger CCPE. Because the instrument is self-reported, scores reflect students' perceived competencies and dispositions rather than directly observed behaviors or performance.

### Data analysis

Data distributions were evaluated for normality using the Kolmogorov–Smirnov test. Baseline group differences in CCPE were tested with independent-samples *t* tests. Intervention effects were examined using a 2 (time: pretest vs. posttest) × 2 (group: intervention vs. control) mixed-design repeated-measures ANOVA to test main effects and, critically, the time × group interaction as the primary inference (Hong et al., 2025). Within-group pre–post changes were evaluated with paired-samples *t* tests as a supplementary description. Given multiple outcomes, *p*-values were interpreted cautiously in conjunction with effect sizes and consistency of patterns across outcomes, acknowledging potential Type I error inflation. Effect sizes (partial η^2^ for ANOVA and Cohen's d for pre–post comparisons) were reported to aid interpretation of practical significance. Assumptions for parametric analyses (e.g., normality and homogeneity of variance) were checked; because the within-subject factor had two levels (pre/post), sphericity was not applicable. Analyses were conducted using complete cases for each outcome in SPSS version 27.0, with two-tailed tests and p < 0.05.

## Results

### Preliminary tests

**Figure 1:**
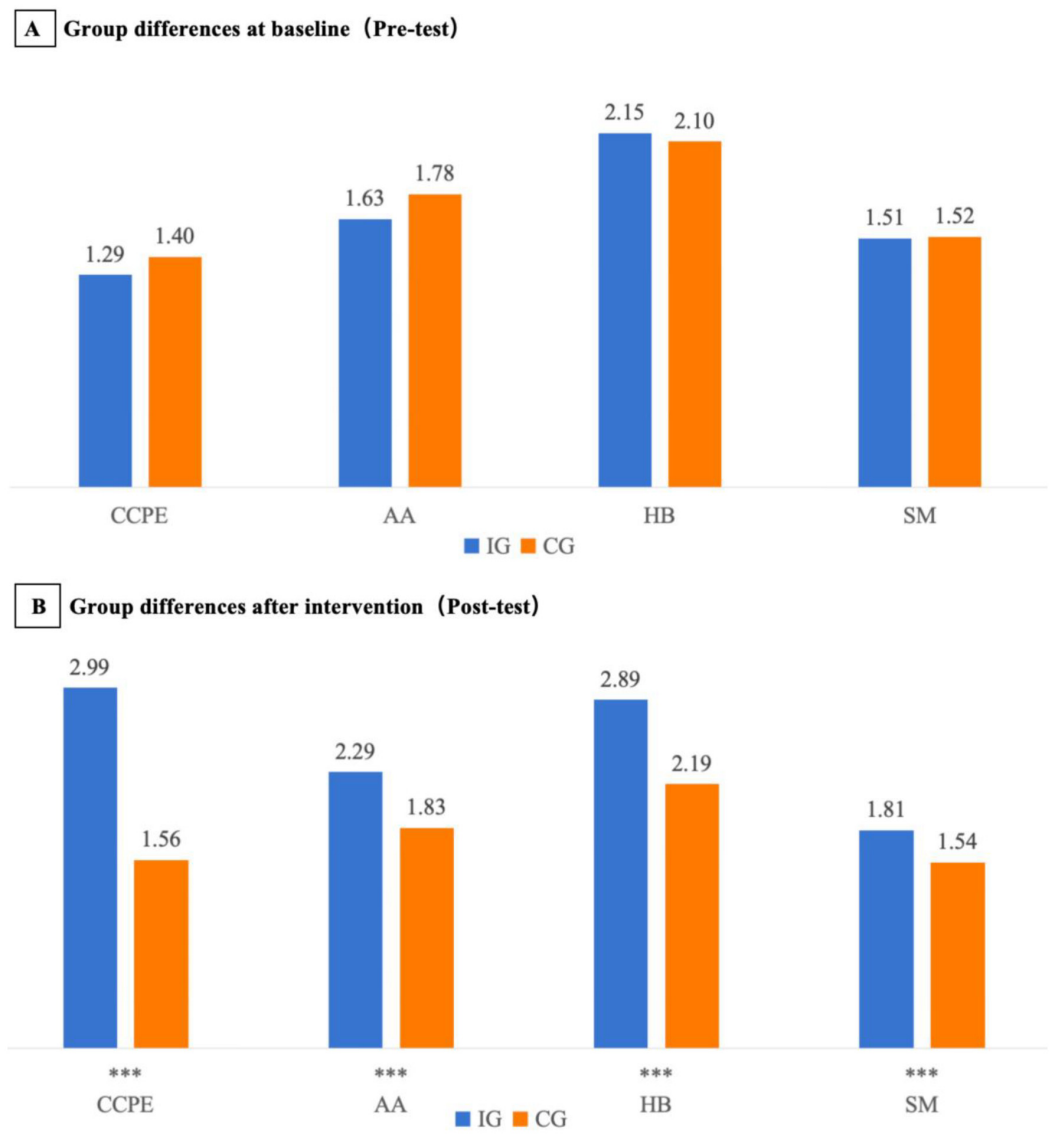
Pre- and post-intervention comparisons between the intervention group (IG) and control group (CG) on CCPE and its subdomains (AA, HB, SM). **(A)** Baseline group comparison. **(B)** Post-test group comparison after the 12-week intervention. Bars represent group means. Asterisks indicate between-group differences at the corresponding time point based on independent-samples t tests (^***^p < 0.001). Overall intervention effects (time × group interactions) were evaluated using a 2 (time) × 2 (group) mixed-design repeated-measures ANOVA (see Table 3). For visualization only, plotted values were offset by subtracting constants (CCPE: 177; AA: 30; HB: 90; SM: 53); this does not affect statistical inference. **Abbreviations:** CCPE, core competencies in physical education; AA, athletic ability; HB, health behaviors; SM, sport morals.

Independent-samples *t* tests indicated no between-group differences at baseline in CCPE (*t* = −0.04, *p* = 0.966), AA (*t* = −0.18, *p* = 0.861), HB (*t* = 0.03, *p* = 0.975), and SM (*t* = −0.01, *p* = 0.989) (see Table 2 and Figure 1). Taken together, the baseline results provide a reasonable basis for evaluating differential pre-post changes between the EPE and conventional PE conditions.

**Table 2:** The baseline levels of CCPE, AA, HB, and SM in both the intervention and control groups before the intervention.

		**Intervention group**		**Control group**			
**Indicator**	**Range**	**Mean**	**SD**	**Mean**	***SD***	***T***	***P***
**CCPE**	1-225	178.29	18.71	178.40	19.04	−0.04	0.966
**AA**	1-45	31.63	6.38	31.78	6.16	−0.18	0.861
**HB**	1-115	92.15	11.48	92.10	11.52	0.03	0.975
**SM**	1-65	54.51	4.79	54.52	4.47	−0.01	0.989

CCPE, core competencies in physical education; AA, athletic ability; HB, health behaviors; SM, sport morals.

### Intervention effects

**Figure 2:**
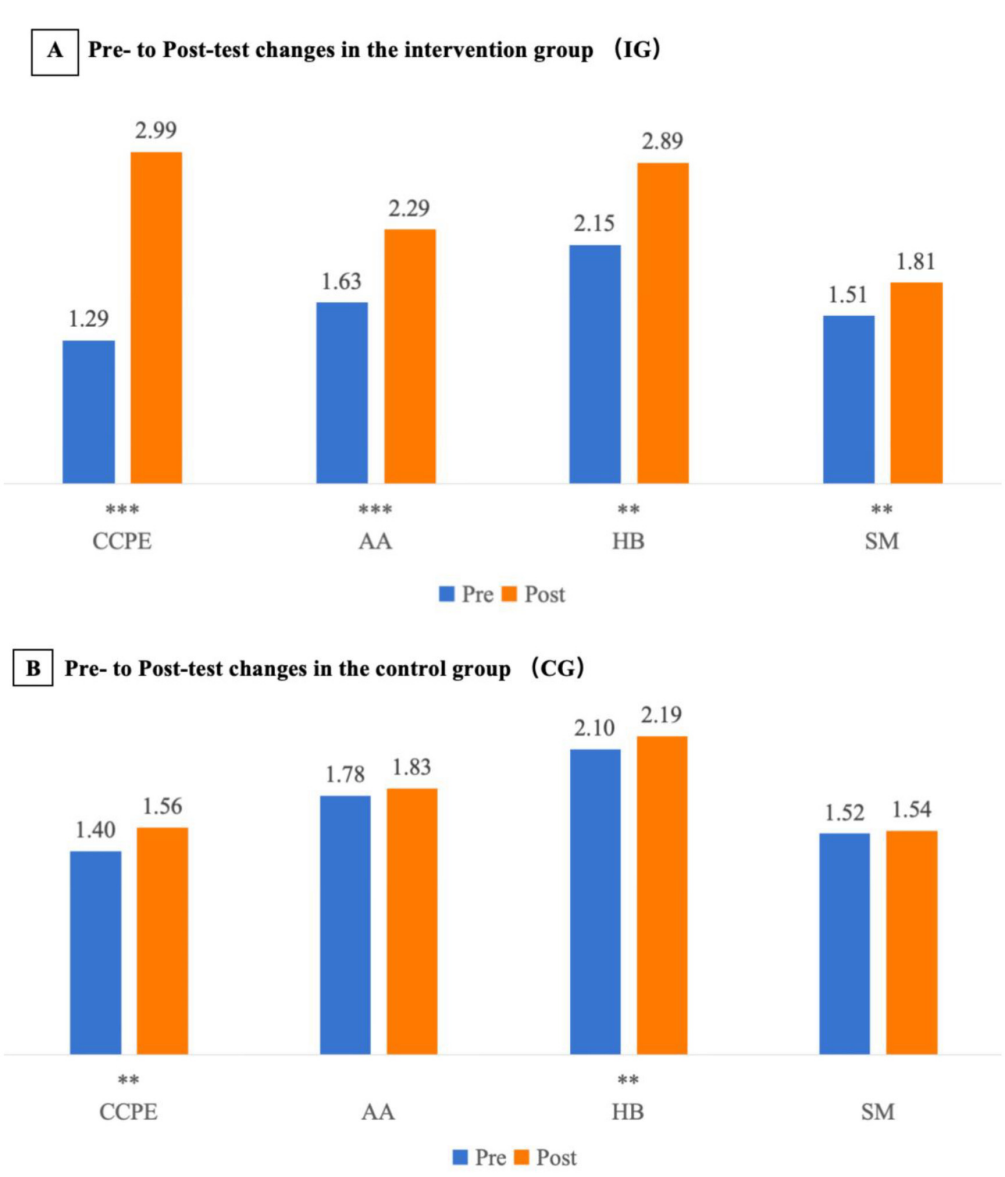
Pre–post trajectories of CCPE and domain scores in the intervention (IG) and control (CG) groups. **(A)** Shows pre- and post-test mean scores for the IG, and **(B)** Shows corresponding means for the CG. The figure is intended to aid interpretation of the intervention effects by visually summarizing the direction and relative magnitude of within-group change and the between-group contrast in change over time (consistent with the time × group interaction tests reported in Table 3). Statistical significance refers to within-group pre–post differences tested with paired-samples t tests (see Supplementary Table 1). Asterisks indicate between-group differences at the corresponding time point based on independent-samples t tests (**p < 0.01, ***p < 0.001). For visualization only, plotted values were offset by subtracting constants (CCPE: 177; AA: 30; HB: 90; SM: 53) to place the series on a comparable visual scale; all statistical analyses were conducted on the original (untransformed) scores. CCPE, core competencies in physical education; AA, athletic ability; HB, health behaviors; SM, sport morals.

Table 3 reports the mixed-design repeated-measures ANOVA for CCPE and its subdomains. Consistent with our study aims, the intervention group demonstrated greater pre–post improvement than the control group in overall CCPE as well as in AA, HB, and SM (i.e., differential gains favoring EPE). There were significant main effects of time for CCPE, AA, HB, and SM (*F* = 15.04–26.57, *p* < 0.001), with corresponding partial η^2^ values ranging from 0.07 to 0.10 (Table 3), indicating overall improvement over time. More importantly, significant time × group interactions were observed for CCPE (*F* = 18.23, *p* < 0.001; partial η^2^ = 0.08), AA (*F* = 16.09, *p* < 0.001; partial η^2^ = 0.08), HB (*F* = 10.13, *p* < 0.01; partial η^2^ = 0.05), and SM (*F* = 11.55, *p* < 0.001; partial η^2^ = 0.06), indicating greater pre–post gains in the intervention group than in the control group. To facilitate interpretation of these interaction effects, Figure 2 provides a visual summary of the pre–post trajectories for CCPE and each domain in the EPE and control groups. For descriptive completeness, paired-samples *t* tests and within-group pre–post effect sizes (Cohen's d) are provided in Supplementary Table 1.

**Table 3:** Results of the repeated-measures ANOVA for CCPE, AA, HB, and SM in the intervention and control groups.

	**Intervention group**		**Control group**		**ANOVA effects (F**, η**p**^**2**^**)**		
**Indicator**	**Pre-test M** ±**SD**	**Post-test M** ±**SD**	**Pre-test M** ±**SD**	**Post-test M** ±**SD**	**Time (F, partial** η^2^**)**	**Group (F, partial** η^2^**)**	Time^*^group (F, partial η^2^**)**
**CCPE**	178.29 ± 18.71	179.99 ± 16.66	178.40 ± 19.04	178.56 ± 18.80	26.57^***^, 0.07	0.07, 0.00	18.23^***^, 0.08
**AA**	31.63 ± 6.38	32.29 ± 5.35	31.78 ± 6.16	31.83 ± 6.05	21.67^***^, 0.10	10.08, 0.00	16.09^***^, 0.08
**HB**	92.15 ± 11.48	92.89 ± 10.53	92.10 ± 11.52	92.19 ± 11.46	16.66^***^, 0.08	24.48, 0.00	10.13^**^, 0.05
**SM**	54.51 ± 4.79	54.81 ± 4.47	54.52 ± 4.47	54.54 ± 4.43	15.04^***^, 0.07	3.55, 0.00	11.55^***^, 0.06

CCPE, core competencies in physical education; AA, athletic ability; HB, health behaviors; SM, sport morals. Effect sizes for ANOVA are reported as partial eta squared (ηp^2^). F values are reported to two decimal places, and ηp^2^ values are reported to two decimal places (values < 0.01 are shown as < 0.01). If ηp^2^ values are displayed as 0.00 in the table due to rounding, they should be interpreted as ηp^2^ < 0.01. Asterisks indicate statistical significance (^*^p < 0.05, ^**^p < 0.01, ^***^p < 0.001). P values for the Group main effect are not shown because the primary inference focuses on the time × group interaction; the Group main effect is reported descriptively.

### Intragroup comparisons

To minimize redundancy with the mixed ANOVA results, within-group paired-samples t tests are reported only in Supplementary Table 1 and are not repeated in the main text (see also Figure 2).

## Discussion

This study examined whether EPE was associated with greater pre-post improvement in high school students' core CCPE compared with conventional PE, using a quasi-experimental parallel-group design. Overall, EPE was associated with statistically significant but modest differential gains in overall CCPE and in each domain (AA, HB, and SM), as indicated by the time × group interactions. These findings suggest that reorganizing PE into interest-based elective modules is feasible under routine school conditions and may support multidimensional CCPE development. However, interpretations should remain cautious given the non-randomized, school-level allocation and the exclusive reliance on self-reported outcomes. Importantly, the observed effects likely reflect small shifts in students' perceived competencies rather than immediate improvements in objectively observed performance or behavior.

### Overall impact of EPE on core competencies in physical education

Aligned with China's PE Curriculum Standards, CCPE in this study was examined across three domains (AA, HB, and SM) (Ministry of Education of the People's Republic of China., 2018, 2022). Accumulating evidence suggests that how PE is organized meaningfully shapes students' learning conditions and competency-related outcomes: compared with conventional class-based delivery, choice-based formats tend to promote engagement and sustained participation, which can translate into more favorable patterns in skill-related and affective indicators (Goyibova et al., 2025; Tran et al., 2024; Coppola et al., 2023). From this perspective, elective models may be particularly well suited to CCPE development because they operationalize differentiated instruction by accommodating heterogeneous interests and needs while remaining curriculum-aligned (Sun and Xiao, 2024).

Consistent with prior evidence, students in the EPE condition showed greater gains in CCPE than those receiving conventional PE. Although mechanisms were not directly assessed, this pattern is consistent with the autonomy-supportive features of EPE: providing meaningful sport choice may better align learning with students' interests, which in turn may encourage more sustained engagement in PE (Fishbach and Woolley, 2022; Holzer et al., 2021). The control group also improved, but to a lesser extent, suggesting that interest- and choice-based instructional organization may offer an incremental advantage for CCPE development under routine school conditions (Alrabai, 2021).

From a SDT perspective, EPE may be associated with CCPE gains through greater support for students' basic psychological needs (Ryan and Deci, 2000). To clarify the theoretical linkage to our domain findings, EPE may (a) support autonomy through interest-based module selection and a stronger sense of ownership; (b) support competence through structured practice, clear goals, and competence-oriented feedback—mechanisms most directly aligned with the observed gains in AA; and (c) support relatedness through stable cross-class elective groupings and cooperative tasks—processes most relevant to the observed gains in SM. These need-supportive experiences may also help students internalize health-oriented values and self-regulatory skills, providing a plausible pathway to the modest improvements observed in HB.

### Impact of EPE on athletic ability

As specified in China's PE Curriculum Standards, AA reflects an integrated form of movement competence—encompassing fitness, sport-specific proficiency, and psychological readiness—and is treated as foundational within the CCPE framework (Ministry of Education of the People's Republic of China., 2018, 2022). In the present study, AA showed a clear differential gain favoring EPE, suggesting an incremental advantage beyond the overall improvement observed over time.

One plausible interpretation is that EPE's fixed enrollment in an interest-aligned sport module creates more sustained opportunities for practice, feedback, and progressive skill development (Qianqian et al., 2023). From an SDT perspective, such a structure can be more autonomy- and competence-supportive: students exercise meaningful choice and are more likely to persist in activities in which they feel capable, which may increase engagement and deliberate practice (Evans et al., 2024; Ryan and Deci, 2000). These conditions are consistent with pathways through which AA may improve under elective, choice-based instruction (Cullen and Oppenheimer, 2024; Ntoumanis and Moller, 2025). By contrast, conventional PE often prioritizes broader content coverage and shorter learning cycles, which may limit sustained, progressive motor learning within a single sport context (Yan et al., 2023).

### Impact of EPE on health behaviors

HB represent students' capacity to maintain and enhance physical and psychological wellbeing and to adapt to environmental demands within China's CCPE framework (Ministry of Education of the People's Republic of China., 2018, 2022). In this study, the intervention group showed greater pre-post improvement in HB than the control group, indicating a modest but consistent differential gain associated with EPE.

A plausible interpretation is that EPE may create conditions conducive to HB development by supporting students' autonomy and competence. SDT suggests that autonomy support facilitates the internalization of health-related values, whereas competence support strengthens confidence to initiate and sustain health-promoting actions (Ryan and Deci, 2000). In parallel, process-oriented models of behavior change propose that students are more likely to translate intentions into action when engagement is high and early mastery experiences build self-efficacy (Marentes-Castillo et al., 2024). Because EPE centers on meaningful choice and sustained participation in an interest-aligned module, it may enhance engagement and provide repeated mastery opportunities, thereby supporting gradual HB improvement (Sheeran et al., 2021). Notably, HB also improved in the control group, underscoring that conventional PE can still contribute to health-related objectives, although the magnitude of change was smaller under routine delivery (Tran et al., 2024; Crotti and Borgogni, 2025).

### Impact of EPE on sport morals

SM capture students' sportsmanship-related norms and value commitments as enacted in sport contexts within China's CCPE framework (Ministry of Education of the People's Republic of China., 2018, 2022). In this study, SM showed a modest but clear differential gain favoring EPE, suggesting that the elective structure may be conducive to students' socio-moral development under routine school conditions.

A plausible interpretation is that EPE strengthens SM by shaping peer norms and relatedness processes. Because students participate in stable, interest-based elective groups, EPE may create repeated opportunities for rule-governed play, role responsibility, and peer feedback—conditions that can support the gradual internalization of sportsmanship norms such as fairness and respect (Kao, 2019; Luguetti et al., 2019). From an SDT perspective, these stable group settings may enhance relatedness and mutual accountability, which can facilitate the internalization of behavioral norms beyond superficial compliance (Ryan and Deci, 2000). This interpretation is consistent with prior work suggesting that structured participation emphasizing rules and responsibility can support moral and social outcomes in PE (D'Isanto et al., 2022).

### Educational implications and practical significance

Although the intervention produced statistically significant gains, the absolute score changes across CCPE domains were modest. These findings should therefore be interpreted cautiously and not framed as immediate, large improvements in students' objective performance or behavior, particularly given the 12-week duration and the self-reported nature of the outcomes (Telford et al., 2021). Nevertheless, small shifts observed under routine school conditions can still be educationally meaningful: they suggest that reorganizing PE into interest-based elective modules may nudge students' perceived competencies in the intended direction without adding instructional time (García-Hermoso et al., 2021). In practice, such small gains may be more realistically interpreted as early-stage movement in students' perceptions that could accumulate with sustained exposure, and they should not be assumed to translate directly into measurable short-term changes in fitness, skill performance, or out-of-class physical activity without additional supports. From an implementation perspective, EPE may thus be a feasible and potentially scalable curricular option, with benefits more likely to accumulate through sustained delivery, ongoing refinement, and alignment with longer-term assessment and monitoring cycles (Bandeira et al., 2022). At the same time, scaling may be constrained by teacher capacity, facilities, and scheduling, which should be considered when interpreting feasibility across school contexts.

### Recommendations for scaling EPE in Chinese high schools

Scaling EPE in Chinese high schools requires attention to several practical conditions. First, teacher professional development should prioritize autonomy-supportive pedagogy (e.g., providing meaningful choice and rationale, and using competence-oriented feedback), alongside modular curriculum planning and strategies for managing mixed-ability elective groups. Training that explicitly integrates and assesses sportsmanship within game-based and cooperative tasks may be particularly important for strengthening SM outcomes (Csordás-Makszin et al., 2025). Second, schools should ensure that elective offerings are feasible within existing facilities and equipment by matching parallel modules to venue capacity and establishing minimum resource benchmarks to support consistent delivery across sports (Bandeira et al., 2022). Third, organizational and scheduling arrangements should be designed to enable cross-class regrouping—through transparent selection rules, balanced group sizes, and mechanisms to resolve timetable conflicts—while maintaining a stable weekly rhythm compatible with examination periods and broader academic demands (Olsen et al., 2021). Finally, scale-up should be accompanied by basic fidelity supports, including standardized teaching plans, periodic peer observation or instructional coaching, and simple process indicators (e.g., attendance, lesson completion, and safety records), to enhance feasibility, equity, and sustainability (Lonsdale et al., 2019).

### Strengths, limitations, and future directions

To our knowledge, this is the first study to examine associations between EPE and CCPE in Chinese high schools using a controlled, pretest–posttest design. The study has two main strengths. First, it addresses a policy-relevant gap by evaluating EPE within China's ongoing curriculum reform and in naturalistic school settings, which enhances ecological validity and practical relevance. Second, the parallel-group comparison (EPE vs. conventional PE), together with standardized contact time, aligned competency targets, and fidelity monitoring, strengthens the interpretability of the observed between-group differences.

Several limitations should be noted. First, the sample was drawn from only two urban public high schools in Chongqing, which limits generalizability beyond similar contexts; replication across regions and school types with larger samples is needed. Second, because EPE involved interest-based sport selection, self-selection bias cannot be ruled out. Although modules were standardized in instructional structure and assessment, unmeasured differences (e.g., baseline motivation or prior sport experience) may have contributed to the observed gains. Notably, despite baseline equivalence on CCPE outcomes, residual confounding at both the student and school levels cannot be excluded. Future studies should incorporate direct measures of motivation and baseline skill/fitness and apply stronger analytic controls to better address residual confounding. Third, all outcomes were self-reported, raising concerns about social desirability and common-method bias, particularly for health behaviors (HB) and sport morals (SM). Future research should triangulate self-report with objective indicators (e.g., accelerometer-based activity or fitness tests) and observational measures (e.g., teacher ratings or structured behavioral observations). Finally, the 12 week intervention did not include follow-up assessments, limiting conclusions about sustainability. Longitudinal or multi-wave designs (e.g., post-test plus 3-, 6-, and 12-month follow-ups or repeated assessments across semesters) are needed to evaluate whether EPE-related gains are maintained, attenuate, or accumulate over time.

## Conclusion

This quasi-experimental study suggests that EPE is associated with modest improvements in high school students' CCPE relative to conventional PE. Given the non-randomized, school-level allocation and voluntary sport choice, these findings should be interpreted as associations rather than definitive causal effects. While SDT offers a plausible explanatory lens, proposed mechanisms were not directly measured. Overall, EPE appears feasible under routine school conditions and warrants further evaluation using longer follow-up and objective or observational outcomes to inform responsible scale-up.

## Data Availability

The raw data supporting the conclusions of this article will be made available by the authors, without undue reservation.
