# Supplementary material for: The impact of elective physical education on high school students' core PE competencies: a quasi-experimental study
Source: Front Psychol. 2026 Mar 26;17:1726417. doi: 10.3389/fpsyg.2026.1726417 (PMC13062215; doi:10.3389/fpsyg.2026.1726417)
Supplement: Supplementary file 1 [file Supplementary_file_1.docx]

Supplementary Material

**Supplementary Table S1.** Differences in CCPE, AA, HB, and SM between the intervention and control groups before and after the intervention.

| **Group** | **Indicator** | **Pre-test**  **M±SD** | **Post-test**  **M±SD** | **Pre-post difference**  **M±SD** | **t** | **Cohen’s d** |
| --- | --- | --- | --- | --- | --- | --- |
| **Intervention Group** | **CCPE** | 178.29±18.71 | 179.99±16.66 | 1.70±3.61 | -4.57*** | 0.47 |
|  | **AA** | 31.63±6.38 | 32.29±5.35 | 0.66±1.51 | -4.22*** | 0.44 |
|  | **HB** | 92.15±11.48 | 92.89±10.53 | 0.74±2.02 | -3.58** | 0.37 |
|  | **SM** | 54.51±4.79 | 54.81±4.47 | 0.30±0.80 | -3.61** | 0.38 |
| **Control Group** | **CCPE** | 178.40±19.04 | 178.56±18.80 | 0.16±0.46 | -3.43** | 0.35 |
|  | **AA** | 31.78±6.16 | 31.83±6.05 | 0.05±0.26 | -1.91 | 0.19 |
|  | **HB** | 92.10±11.52 | 92.19±11.46 | 0.09±0.29 | -3.13** | 0.31 |
|  | **SM** | 54.52±4.47 | 54.54±4.43 | 0.02±0.20 | -1.00 | 0.10 |

**Note:** CCPE = core competencies in physical education; AA = athletic ability; HB = health behaviors; SM = sport morals. Effect sizes for within-group pre–post changes are reported as Cohen’s d. Asterisks indicate statistical significance (*p < .05, **p < .01, ***p < .001).

**Supplementary Table S2.** Item-level pre-test and post-test descriptive statistics (M ± SD) to support interpretation of domain-level changes in CCPE (AA, HB, SM) across groups.

| **Indicator** | **Statement** | **Intervention Group** | | **Control Group** | |
| --- | --- | --- | --- | --- | --- |
|  |  | **Pre-test**  **M±SD** | **Post-test**  **M±SD** | **Pre-test**  **M±SD** | **Post-test**  **M±SD** |
| AA | I have mastered the basic techniques and tactics of the sports I have learned. | 3.80±0.99 | 4.05±0.68 | 3.61±0.69 | 3.61±0.71 |
| AA | I have mastered the competition rules of the sports I have learned. | 3.81±0.92 | 3.88±0.77 | 4.05±0.84 | 4.06±0.82 |
| AA | I understand methods for developing exercise prescriptions. | 3.30±1.17 | 3.44±0.95 | 3.36±1.10 | 3.36±1.10 |
| AA | I am able to apply the sports I have learned to daily exercise or competitions. | 3.79±0.85 | 3.79±0.82 | 3.72±0.80 | 3.73±0.77 |
| AA | I am aware of major domestic and international sports events related to the sports I have learned. | 3.37±0.88 | 3.41±0.81 | 3.41±0.86 | 3.42±0.86 |
| AA | I can analyze important events and issues that occur in sports competitions. | 3.37±0.89 | 3.38±0.83 | 3.40±0.94 | 3.40±0.94 |
| AA | I can develop physical fitness exercise plans for family members or classmates. | 3.13±0.96 | 3.20±0.82 | 3.13±1.06 | 3.13±1.06 |
| AA | I am able to adjust my own physical fitness exercise plan based on workout results. | 3.43±0.99 | 3.47±0.90 | 3.46±0.96 | 3.46±0.96 |
| AA | I can correctly evaluate the effects of physical fitness exercise. | 3.64±0.80 | 3.66±0.74 | 3.65±0.77 | 3.67±0.76 |
| HB | I am able to proactively participate in or organize sports competitions within my class. | 3.67±0.96 | 3.83±0.71 | 3.73±0.89 | 3.72±0.92 |
| HB | I know that the positive emotions generated by physical exercise far outweigh the negative ones. | 4.06±0.84 | 4.10±0.78 | 4.05±0.79 | 4.06±0.78 |
| HB | I persist in exercising the sports I like. | 3.73±0.93 | 3.77±0.86 | 3.78±0.96 | 3.80±0.91 |
| HB | I have good physical exercise habits. | 3.61±0.99 | 3.68±0.87 | 3.64±0.98 | 3.64±0.98 |
| HB | Even without Physical Education exams, I would still persist with physical exercise. | 3.64±0.96 | 3.69±0.87 | 3.70±0.97 | 3.72±0.93 |
| HB | I maintain good personal and public hygiene habits. | 4.45±0.73 | 4.47±0.65 | 4.37±0.74 | 4.37±0.74 |
| HB | I never litter and can sort and dispose of garbage properly. | 4.13±0.79 | 4.15±0.72 | 4.14±0.80 | 4.14±0.80 |
| HB | I know the characteristics and patterns of psychological development during adolescence. | 3.82±0.88 | 3.86±0.80 | 3.82±0.90 | 3.82±0.90 |
| HB | I understand the harm of malnutrition to physical health. | 4.04±0.76 | 4.05±0.74 | 4.04±0.72 | 4.04±0.72 |
| HB | I understand that different intensities of exercise require different nutrition. | 3.80±0.91 | 3.88±0.76 | 3.92±0.83 | 3.92±0.83 |
| HB | I understand the dangers, transmission routes, and preventive measures of infectious diseases. | 4.00±0.67 | 4.01±0.63 | 4.02±0.70 | 4.02±0.70 |
| HB | I actively do my best to prevent various diseases. | 4.19±0.75 | 4.20±0.71 | 4.16±0.74 | 4.16±0.74 |
| HB | I possess safety awareness and capabilities. | 4.10±0.59 | 4.10±0.59 | 4.11±0.60 | 4.11±0.60 |
| HB | I have a relatively comprehensive grasp of methods for self-protection and mutual protection during sports. | 4.07±0.69 | 4.11±0.63 | 4.09±0.68 | 4.09±0.68 |
| SM | I have good health awareness and focus on developing a healthy and civilized lifestyle. | 4.09±0.65 | 4.10±0.61 | 4.13±0.66 | 4.13±0.66 |
| SM | I understand the harm of negative emotions to health. | 4.28±0.78 | 4.30±0.73 | 4.18±0.78 | 4.18±0.78 |
| SM | I have a positive and optimistic attitude towards life. | 4.18±0.70 | 4.18±0.70 | 4.15±0.72 | 4.15±0.72 |
| SM | I know that depression is a negative emotion. | 4.29±0.62 | 4.30±0.60 | 4.25±0.59 | 4.25±0.59 |
| SM | I am able to distinguish between positive and negative emotions. | 4.16±0.79 | 4.17±0.76 | 4.03±0.81 | 4.03±0.81 |
| SM | I have good social interaction skills. | 4.11±0.73 | 4.13±0.68 | 4.05±0.65 | 4.05±0.65 |
| SM | I can adapt relatively quickly to new learning and living environments. | 3.78±1.00 | 3.82±0.92 | 3.76±1.01 | 3.76±1.01 |
| SM | After entering a new class, I take the initiative to invite classmates to exercise together. | 4.12±0.73 | 4.13±0.74 | 4.13±0.66 | 4.18±0.62 |
| SM | I know that the harmonious blending of competition and cooperation helps me progress faster. | 4.09±0.74 | 4.14±0.68 | 4.01±0.68 | 4.00±0.69 |
| SM | When encountering opponents stronger than me in competitions, I dare to accept the challenge. | 4.02±0.84 | 4.09±0.74 | 3.98±0.81 | 3.99±0.78 |
| SM | In sports activities, I can face difficulties directly and am not afraid of them. | 4.02±0.86 | 4.04±0.79 | 4.05±0.72 | 4.05±0.71 |
| SM | When encountering problems in physical education learning, I actively seek guidance from the teacher. | 4.21±0.82 | 4.22±0.78 | 4.16±0.75 | 4.16±0.75 |
| SM | Even after getting a full score in a physical education test, I am still willing to continue learning and practicing. | 4.40±0.66 | 4.41±0.61 | 4.38±0.60 | 4.39±0.60 |
| SM | I am able to consciously abide by competition rules. | 4.37±0.72 | 4.40±0.63 | 4.39±0.65 | 4.39±0.65 |
| SM | During physical fitness tests, I demonstrate integrity and do not engage in cheating. | 4.43±0.70 | 4.44±0.65 | 4.38±0.60 | 4.38±0.60 |
| SM | When the teacher selects members for the school sports meet, I participate in the competition fairly. | 4.41±0.58 | 4.41±0.58 | 4.35±0.71 | 4.36±0.72 |
| SM | In physical activities, I take care of sports equipment and actively assist the teacher in setting up the venue and organizing equipment. | 4.35±0.48 | 4.35±0.48 | 4.35±0.48 | 4.35±0.48 |
| SM | In physical activities, I get along well with classmates and respect others. | 4.00±0.59 | 4.01±0.60 | 4.06±0.66 | 4.06±0.66 |
| SM | I have a good sense of teamwork and can practice cooperatively with classmates. | 3.83±0.56 | 3.83±0.56 | 3.95±0.53 | 3.95±0.53 |
| SM | In competitions, for the ultimate victory of the team, I am willing to create better scoring opportunities for my teammates. | 4.07±0.51 | 4.09±0.50 | 4.17±0.47 | 4.17±0.47 |
| SM | When my own mistakes cause disadvantages for the team, I can actively take responsibility and take remedial measures. | 4.30±0.75 | 4.37±0.66 | 4.28±0.74 | 4.28±0.74 |
| SM | Even if I lose a competition, I do not get discouraged. | 3.85±0.83 | 3.88±0.77 | 3.86±0.83 | 3.86±0.83 |

**Note:** CCPE = core competencies in physical education; AA = athletic ability; HB = health behaviors; SM = sport morals.

**Core Competencies in Physical Education Evaluation Scale for High School Students**

Dear student,

Hello! The following list contains statements related to your participation in physical activity and your health. Please read each item carefully and indicate, based on your specific situation, the extent to which you agree or disagree with each statement. Please mark "√" on the number that best matches your opinion for each item. When answering each question, please use the following scale:

**1 = Strongly Disagree 2 = Disagree 3 = Somewhat Agree 4 = Agree 5 = Strongly Agree**

The results of this questionnaire are for scientific research purposes only and are not related to your Physical Education grades. Please answer truthfully according to your own thoughts. Thank you for your cooperation!

**School:** Chongqing Bashu High School / Chongqing Dazu Chengnan High School

**Gender:** Male / Female

**Grade:** Grade 10 / Grade 11 / Grade 12

**Class:** _________________________

**Elective Sport Module:** _________________________

**Class Frequency (Number of sessions per week, 40 or 45 minutes each):** _________________________

**Date:** _________________________

| **Indicator** | **Statement** | **Response Scale** | | | | |
| --- | --- | --- | --- | --- | --- | --- |
|  |  | **1 Strongly Disagree** | **2 Disagree** | **3 Somewhat Agree** | **4 Agree** | **5 Strongly Agree** |
| AA | I have mastered the basic techniques and tactics of the sports I have learned. |  |  |  |  |  |
| AA | I have mastered the competition rules of the sports I have learned. |  |  |  |  |  |
| AA | I understand methods for developing exercise prescriptions. |  |  |  |  |  |
| AA | I am able to apply the sports I have learned to daily exercise or competitions. |  |  |  |  |  |
| AA | I am aware of major domestic and international sports events related to the sports I have learned. |  |  |  |  |  |
| AA | I can analyze important events and issues that occur in sports competitions. |  |  |  |  |  |
| AA | I can develop physical fitness exercise plans for family members or classmates. |  |  |  |  |  |
| AA | I am able to adjust my own physical fitness exercise plan based on workout results. |  |  |  |  |  |
| AA | I can correctly evaluate the effects of physical fitness exercise. |  |  |  |  |  |
| HB | I am able to proactively participate in or organize sports competitions within my class. |  |  |  |  |  |
| HB | I know that the positive emotions generated by physical exercise far outweigh the negative ones. |  |  |  |  |  |
| HB | I persist in exercising the sports I like. |  |  |  |  |  |
| HB | I have good physical exercise habits. |  |  |  |  |  |
| HB | Even without Physical Education exams, I would still persist with physical exercise. |  |  |  |  |  |
| HB | I maintain good personal and public hygiene habits. |  |  |  |  |  |
| HB | I never litter and can sort and dispose of garbage properly. |  |  |  |  |  |
| HB | I know the characteristics and patterns of psychological development during adolescence. |  |  |  |  |  |
| HB | I understand the harm of malnutrition to physical health. |  |  |  |  |  |
| HB | I understand that different intensities of exercise require different nutrition. |  |  |  |  |  |
| HB | I understand the dangers, transmission routes, and preventive measures of infectious diseases. |  |  |  |  |  |
| HB | I actively do my best to prevent various diseases. |  |  |  |  |  |
| HB | I possess safety awareness and capabilities. |  |  |  |  |  |
| HB | I have a relatively comprehensive grasp of methods for self-protection and mutual protection during sports. |  |  |  |  |  |
| SM | I have good health awareness and focus on developing a healthy and civilized lifestyle. |  |  |  |  |  |
| SM | I understand the harm of negative emotions to health. |  |  |  |  |  |
| SM | I have a positive and optimistic attitude towards life. |  |  |  |  |  |
| SM | I know that depression is a negative emotion. |  |  |  |  |  |
| SM | I am able to distinguish between positive and negative emotions. |  |  |  |  |  |
| SM | I have good social interaction skills. |  |  |  |  |  |
| SM | I can adapt relatively quickly to new learning and living environments. |  |  |  |  |  |
| SM | After entering a new class, I take the initiative to invite classmates to exercise together. |  |  |  |  |  |
| SM | I know that the harmonious blending of competition and cooperation helps me progress faster. |  |  |  |  |  |
| SM | When encountering opponents stronger than me in competitions, I dare to accept the challenge. |  |  |  |  |  |
| SM | In sports activities, I can face difficulties directly and am not afraid of them. |  |  |  |  |  |
| SM | When encountering problems in physical education learning, I actively seek guidance from the teacher. |  |  |  |  |  |
| SM | Even after getting a full score in a physical education test, I am still willing to continue learning and practicing. |  |  |  |  |  |
| SM | I am able to consciously abide by competition rules. |  |  |  |  |  |
| SM | During physical fitness tests, I demonstrate integrity and do not engage in cheating. |  |  |  |  |  |
| SM | When the teacher selects members for the school sports meet, I participate in the competition fairly. |  |  |  |  |  |
| SM | In physical activities, I take care of sports equipment and actively assist the teacher in setting up the venue and organizing equipment. |  |  |  |  |  |
| SM | In physical activities, I get along well with classmates and respect others. |  |  |  |  |  |
| SM | I have a good sense of teamwork and can practice cooperatively with classmates. |  |  |  |  |  |
| SM | In competitions, for the ultimate victory of the team, I am willing to create better scoring opportunities for my teammates. |  |  |  |  |  |
| SM | When my own mistakes cause disadvantages for the team, I can actively take responsibility and take remedial measures. |  |  |  |  |  |
| SM | Even if I lose a competition, I do not get discouraged. |  |  |  |  |  |
